# Experts’ Willingness to Restore With Direct Composite: Complexity, Cuspal Coverage, and Techniques

**DOI:** 10.3290/j.jad.c_2709

**Published:** 2026-06-04

**Authors:** Oliver Bailey, Frank Pfefferkorn, Thomas Attin, Roland Frankenberger, Bas Loomans, Anne-Katrin Lührs, Nicola Scotti, Diana Wolff, Reinhard Hickel, Niek Opdam

**Affiliations:** a Oliver Bailey Clinical Lecturer, School of Dental Sciences, Newcastle University, Newcastle upon Tyne, UK. Contributed to the idea, methods, analysis, and critically evaluated the manuscript; provided the initial manuscript draft.; b Frank Pfefferkorn Senior Clinical Research Manager, Dentsply Sirona, Clinical Affairs & Workflows, Dentsply DeTrey, DeTrey-Str. 1, 78467 Konstanz, Germany. Contributed to the idea, methods, analysis, and critically evaluated the manuscript.; c Thomas Attin Chairman, Clinic of Conservative and Preventive Dentistry, Center for Dental Medicine, University of Zurich, Plattenstr. 11, CH-8032 Zurich, Switzerland. Contributed to the idea, methods, analysis, and critically evaluated the manuscript.; d Roland Frankenberger Professor and Chair, Department of Operative Dentistry, Endodontics, and Pediatric Dentistry, University Dental Medicine, University of Marburg and University Hospital Giessen and Marburg, Campus Marburg, Georg Voigt St 3, D-35039 Marburg, Germany. Contributed to the idea, methods, analysis, and critically evaluated the manuscript.; e Bas Loomans Professor, Chair of Oral Function and Restorative Dentistry, Radboud University Medical Center, Research Institute for Medical Innovation, Department of Dentistry, Philips van Leydenlaan 25, 6525EX Nijmegen, the Netherlands. Contributed to the idea, methods, analysis, and critically evaluated the manuscript.; f Anne-Katrin Lührs Dentist, Hannover Medical School, Department of Conservative Dentistry, Periodontology and Preventive Dentistry, Carl-Neuberg-Straße 1, Hannover 30625, Germany. Contributed to the idea, methods, analysis, and critically evaluated the manuscript.; g Nicola Scotti Professor and Dean, Dental School Lingotto, University of Via Nizza, 230 – 10126, Turin, Italy. Contributed to the idea, methods, analysis, and critically evaluated the manuscript.; h Diana Wolff Medical Director of the Department of Conservative Dentistry, Medical Faculty, Heidelberg University, Im Neuenheimer Feld 400, 69117 Heidelberg, Germany. Contributed to the idea, methods, analysis, and critically evaluated the manuscript.; i Reinhard Hickel Emeritus Dean of Dental School and Medical Faculty, University Hospital, Ludwig-Maximilians University of Munich, Goethestr. 70 80336 Munich, Germany. Contributed to the idea, methods, analysis, and critically evaluated the manuscript.; j Niek Opdam Retired Assistant Professor, Radboud University Medical Center, Department of Dentistry Nijmegen, The Netherlands. Wateringen 195, 5236 SN Den Bosch, the Netherlands. Contributed to the idea, methods, analysis and critically evaluated the manuscript.

**Keywords:** adhesive dentistry, class II restoration, deep margin elevation, direct composite resin restorations

## Abstract

**Purpose:**

Minimal evidence exists on how class II cavity complexity influences direct restoration provision or if complexifying factors influence the use of techniques and materials. This study aimed to explore: (1) experts’ willingness to restore a variety of specific presentations of class II cavities with direct composite restorations; (2) the influence of cavity characteristics on this and the provision of cuspal coverage; and (3) how varying cavity presentations affected technique and material use.

**Methods and Materials:**

An e-questionnaire developed by international experts was distributed to 67 experts on direct composite restoration. The questionnaire section reported here presented vignettes of varying class II cavities, coded by potential complexifying factors. Respondents were asked if they would restore them with a direct composite. If so, it then asked what specific materials and techniques would be used. Regression analyses were performed.

**Results:**

Vignette response rate was 78%. There was a large variation in willingness to restore varying class II cavity presentations with direct composite. This was significantly influenced by the presence of large inter-proximal “bridging” gaps, root fillings, broad boxes, ≥ 3 cavity surfaces involved and deep sub-gingival margins. When present, these complexifying characteristics affected material and technique use. Those willing to restore more complex cavities were more likely to use more curved circumferential bands and less likely to use a rubber dam, among other factors.

**Conclusion:**

Cavity complexity affects experts’ willingness to place direct composite restorations. The willingness to use a variety of techniques was associated with a greater readiness to restore more complex class II cavities with direct composite.

Class II cavities show significant variation in their presentation. Guidance produced by the European Section of the Academy of Operative Dentistry suggested broad indications for and general technical guidance on the use of direct posterior composites. It did not provide guidance on techniques specific to the varying presentations of class II restorations in any detailed way however.^22^ Class II cavity characteristics, such as the cervical extension in relation to the gingivae, extent of tooth structure loss, and the distance to an adjacent tooth (bridging gap) for example, can increase the complexity of a direct restoration, potentially requiring more steps, equipment, and techniques to restore the cavity effectively.^3,5,11,17,20,28^ Clinicians have also been shown to be less confident in providing direct composite restorations under some of these difficult circumstances,^6,21^ which might influence their willingness to place direct composite restorations. Indirect restorations are commonly recommended as the superior or more durable option for posterior root-filled teeth, and vital teeth with larger cavities and significant biomechanical compromise, eg,^7,27,30–33^ but are significantly more expensive and time-consuming.^4^ This guidance may prevent the pragmatic direct restoration of compromised teeth by dentists, which preferentially affects patients who are less able to pay. Potential sequelae of this are more teeth being extracted, which may be exacerbated by the global phase-down and phase-out of amalgam, and a potential widening of existing health inequalities.^4^ Also, when considering the outcomes of systematic reviews, the claim that indirect restorations have superior durability is not based on robust scientific evidence.^24^ Recent systematic reviews show similar clinical survival of direct and indirect restorations on vital and non-vital teeth^8,15^ and a current German S3 clinical practice guideline considers direct composite a viable option for the restoration of defects involving cusp replacement.^34^ Therefore, alongside data analysis on survival of direct posterior composites,^9^ there is no conclusive evidence that the material and restoration type are major factors limiting restoration survival. This gives space to the theory that for many indications, a large direct restoration could be preferred over an indirect restoration.

Many of the recommendations, alongside recommendations for cuspal coverage (which vary), are often largely based on laboratory studies investigating the biomechanical behavior of compromised teeth and teeth restored in different ways.^2,29,30^ The clinical significance of these findings is uncertain however, as clinical trial data on direct restorations tend to focus on the impact of materials when restoring simple cavities with limited follow-up^26^ and though longevity studies on more extensive adhesive direct restorations exist,^13,14,25^ the data is limited. Characteristics of cavities that make them complex to restore have not been well defined. When confronted with varying presentations of class II cavities, clinicians may therefore take different approaches to (1) treatment planning in terms of providing direct or indirect restorations, and (2) restoring the cavities (when selecting a direct approach) in terms of materials, equipment, and techniques.

The objectives of this study were therefore to understand and quantify (1) experts’ willingness to restore a variety of specific presentations of class II cavities with direct composite restorations, (2) the influence of cavity characteristics on this and the provision of cuspal coverage, and (3) how varying cavity presentations affect technique and material use. The overall aim, allied with data on the general frequency of use of materials and techniques presented in a subsequent paper, was to see if the data could support an expert consensus (where possible with acceptable variations) on the direct restoration of class II cavities with composite resin.

## METHODS AND MATERIALS

**Fig 1:**
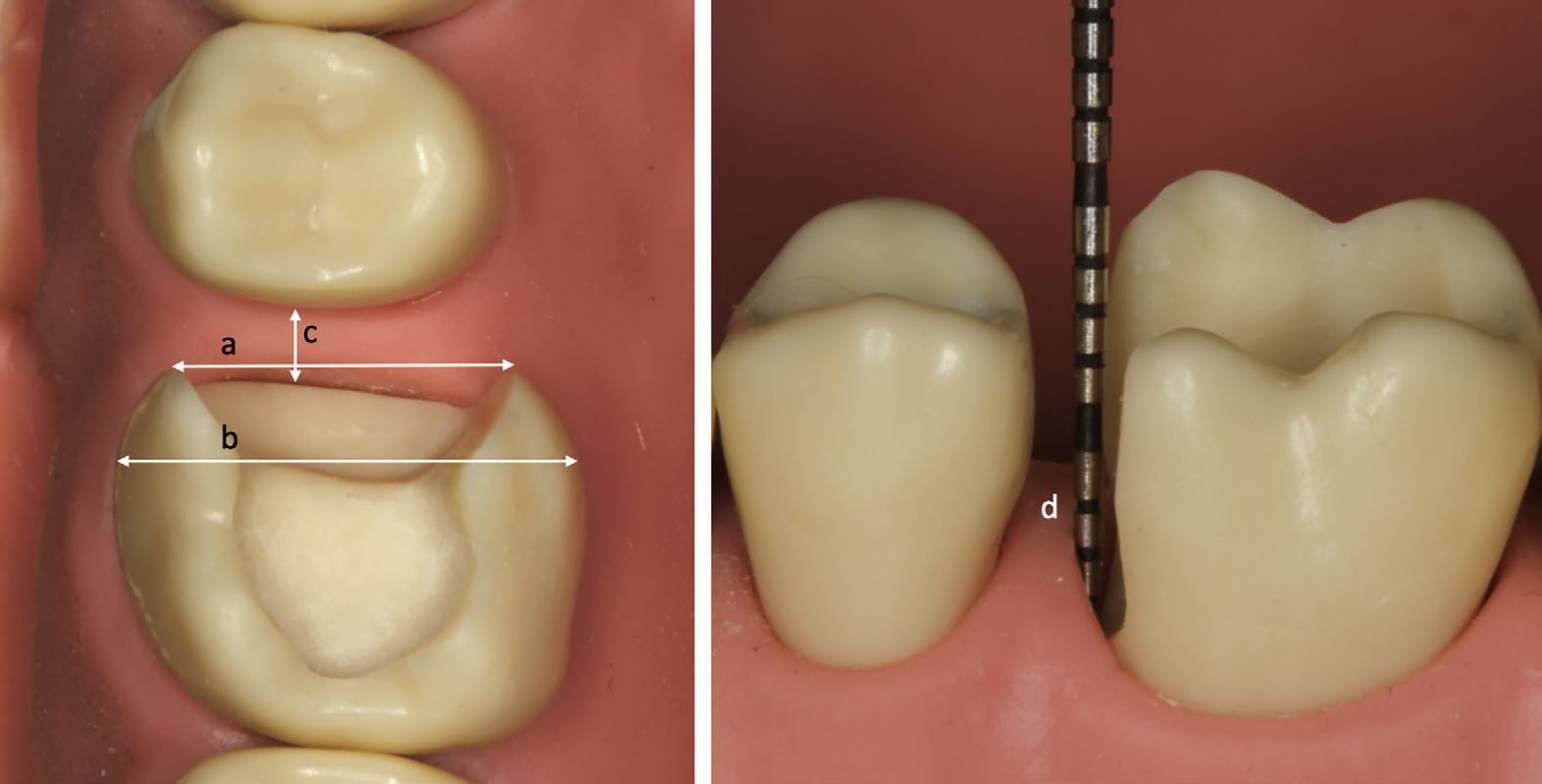
Representation of select cavity variables. a = width of box; b = width of intact tooth; a/b ≥ 0.75 therefore a = broad box; c = large interproximal bridging gap (≥ 2 mm); d = deep sub-gingival cavity margin (≥ 2 mm sub-gingival).

An initial outline of the research proposal was sent to an expert panel of nine restorative dentists from five different countries (Germany, the Netherlands, Switzerland, Italy, UK) (selected through having published research on restorative dentistry, and having a known interest in extensive direct posterior composite restorations). Potentially relevant clinical variables and different class II cavity presentations that might impact their technical management were suggested. Feedback was provided by the panel and collated, leading to the development of a questionnaire that included multiple clinical vignettes using photographs of varying class II cavities to address these questions. The first version of the survey was piloted and answered anonymously by all members of the expert panel, with anonymous feedback also provided. Feedback and initial findings were used as a basis for discussion at two in-person workshops at the combined conference of The European Federation of Conservative Dentistry (EFCD) and The Organization for Caries Research (ORCA), July 2023 in Egmond aan Zee (NL). Changes were made to the wording and structure of the survey, and aims and objectives were defined. Relevant clinical variables which might affect restorative technique were discussed, agreed, and defined. These were later modified slightly to the variables given in Table 1 (justified later) with explanatory pictures in Figure 1.

**Table 1:** Clinical variables assessed

Clinical variable	Options
Adjacent cavities	Yes; No
Adjacent tooth	Yes; No
Root filled	Yes; No
Single surface “claustrophobic” restoration*	Yes; No
≥ Three surface restoration	Yes; No
Broad box**	Yes; No
Large interproximal “bridging gap”^#^	Yes; No
Deep sub-gingival^##^	Yes; No
Thin cusp^§^	Yes; No
*Small and narrow cavity with maximum central extension 3 mm from adjacent contact; **cavity ≥ 3/4 width of intact tooth; ^#^distance from base of cavity to maximum bulbosity of adjacent tooth ≥ 1.5 mm; ^##^cavity margin ≥ 2 mm sub-gingival; ^§^≤ 2 mm remaining cusp wall.	

Further potential cases to be included as vignettes were collected. Vignette inclusion was subsequently determined by two iterative anonymous surveys of the expert group, asking if each proposed vignette should be included. The vignettes were presented with coding on the clinical variables defined in Table 1, and the opportunity for anonymous disagreement was given. Respondents could answer “Yes,” “No,” or “Maybe” to each vignette’s inclusion and had the opportunity to comment on each vignette. The overall decision on inclusion was taken following discussion between OB and NO bearing in mind the expert responses and study objectives to present a broad range of class II cavities with differing characteristics. An example of the coding of a vignette used in the questionnaire is included in Appendix Figure A1. Final piloting to the expert group resulted in no further changes.

The study is reported to be consistent with STROBE (Strengthening the Reporting of Observational Studies in Epidemiology) guidelines. A favorable ethical opinion was obtained from Newcastle University Ethics Committee (ref: 30898/2022). The e-questionnaire was built with a contracted version of SurveyMonkey software (https://www.surveymonkey.com/) to allow answer-driven question options and complex logic between questions. Participation was voluntary, with consent required for admission to the survey, and responses were anonymized. A PDF version of the survey is available in Supplementary Information 1. It had two sections, one assessing the general frequency of material and technique use, which is reported in a subsequent paper, and the other provided participants with 15 specific clinical photographic vignettes showing 19 cavities. It asked first if they would restore the tooth (or teeth, where adjacent cavities were present) with a direct composite restoration. If so, further questions were asked, including whether cuspal coverage would be provided and which equipment, materials, and techniques respondents would use for the specific cavity. Demographic information relating to experience, country of graduation, and practice was obtained at the end of the survey.

### Sample

The core expert group suggested clinicians known to them who were performing direct restorations to a high standard and with an interest in the area. They were approached by the core group to indicate their willingness to participate, and a closed sampling frame with a convenience sample of 67 experts was invited through personalized email invitations. This aimed to provide sufficient data and group sizes to allow for logistic regression analyses. The survey briefly explained the purpose of the study before obtaining consent to participate. Two email reminders were sent. The survey was launched on September 18, 2024, and the deadline for responses was November 8, 2024.

### Data Analysis

The raw data collected anonymously by SurveyMonkey were forwarded to the research group. The dataset was cleaned and merged with the coded cavity characteristics of each presented vignette. Data were analyzed descriptively and converted from wide to long format to allow logistic regression alongside descriptive statistical analysis in Stata software (version 18; StataCorp MP). Respondent demographic groups were defined, and potentially influential variables were listed and agreed upon by the core group for each analysis.

Variables with low case representation were omitted (concavity) or combined (missing numbers of cusps), as was the premolar-molar variable, because the other cavity characteristics were not evenly distributed between the different tooth types, all of which could have potentially resulted in misleading outcomes.

The following logistic regression analyses were explored:

How cavity characteristics (including interaction effects) influenced the willingness to place a direct class II composite restoration and how they influenced the provision of cuspal coverage.
How techniques changed as relevant cavity characteristics varied
What influenced the use of a rubber (dental) dam
How operators’ willingness to place direct composite restorations influenced techniques used.

Collinearity was assessed by calculating variance inflation factors (VIFs) and correlation matrices. All model VIFs were less than 3.5, suggesting minimal collinearity, aside from where interaction effects were included and explored. These were highly correlated and therefore removed. The best model was selected when the lowest Bayesian Information Criterion (BIC) value was obtained using backward stepwise elimination of high *P* value variables.

## RESULTS

Fifty-two respondents answered vignette questions, giving a response rate of 78%, with 44 answering them all, and the final demographic question gave a completion rate of 85% for this section.

### Demographics

Demographic data is presented in Table 2. Groupings of private-only practitioners versus those working totally or partially in academia, and a case-mix of primarily restored teeth versus primarily primary caries, and a fairly even mix were made.

**Table 2:** Respondent demographics

Respondent demographics		Percentage (%)
Years qualified (n = 42)	< 10	5
	10 – < 15	12
	15 – < 20	29
	20 +	55
Practice (n = 44)	Academic	32
	Academic and private	32
	Private general*	23
	Private specialist	14
Class II restoration case-mix (n = 44)	Primarily primary caries	10
	Primarily restored teeth**	30
	Fairly even mix	61
Country of practice^#^ (n = 43)	Germany	30
	UK	16
	Italy	14
	Netherlands	12
	Switzerland	12
	Norway	5
	China	2
	Croatia	2
	Iraq	2
	Latvia	2
	Sweden	2
*One respondent stating practice limited to endodontics thus classified; **two respondents stating all teeth root filled thus categorized; ^#^country of practice and country of graduation did not differ for any respondents where recorded.		

### Experts’ Willingness to Restore, Provision of Cuspal Coverage, and Use of Matrices and Rubber Dam Isolation

**Fig 2:**
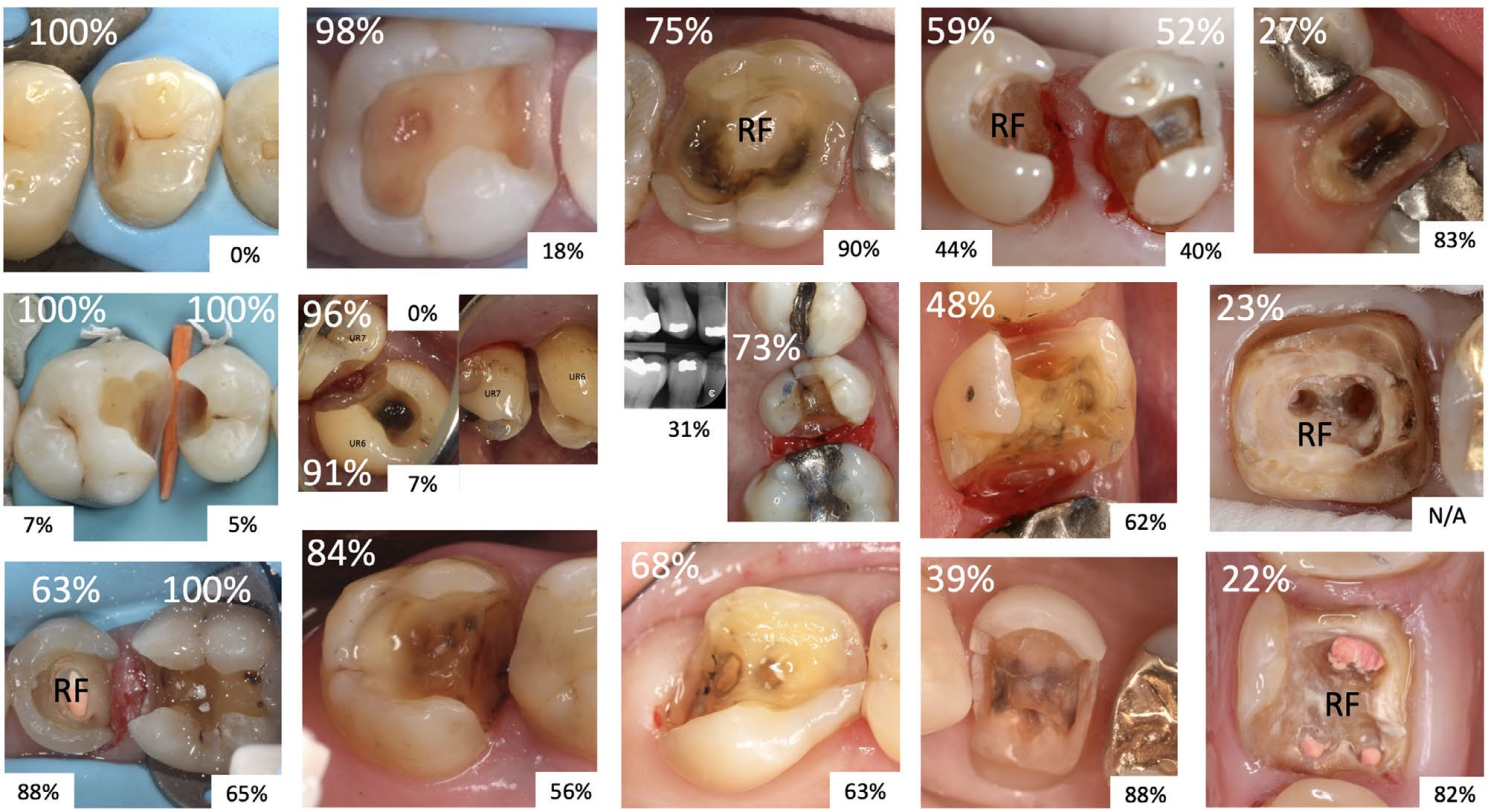
Willingness to restore various cavities and cuspal coverage. Larger white font: percentage of respondents willing to restore the cavity with direct composite; smaller black font: percentage of respondents willing to restore the cavity who would cover cusps; RF, root filled.

Details of cases answered, willingness to restore with direct composite, cuspal coverage, matrix, and rubber dam (not split dam) use by individual respondent are shown in Appendix Table A1. Experts’ willingness to restore the various cavity presentations varied considerably by respondent, with an average of 69% of cavities restored, a standard deviation of 24 and a range of 21–100%. It also varied by cavity, with the percentage of respondents willing to restore each vignette shown in Figure 2. The figure also shows the percentage of those who were willing to restore the cavity who would provide cuspal coverage. The sample was split into those willing to restore ≥ 80% (n = 19) and those who were not for further analysis (Appendix Table A1).

When willing to restore, respondents would provide cuspal coverage in 40% of cavities on average, with a standard deviation of 24 and a range of 0–100%. A small percentage of respondents (6%) never provided cuspal coverage, but they would directly restore fewer than 60% of cavities presented (Appendix Table A1). The two respondents who always covered cusps left the survey after giving responses for 4 and 9 cavities out of 19. Respondents covered cusp(s) of root-filled teeth 83% of the time, mesio-occluso-distal (MOD) or larger cavities 77%, and cavities with thin cusp walls 74% of the time.

Seven respondents (13%) were willing to restore all cases with a direct composite. Six of these never used rubber dam, and the seventh used rubber dam in 88% of cases. Five of the seven respondents used both circumferential matrices and sectional matrices in combination.

Twelve respondents (23%) always used sectional matrices. Eight of these always used rubber dam (15% of the total sample) and eight were willing to restore under 60% of cases with direct composite. Twenty respondents (38%) always used rubber dam. Seven of these always used sectional matrices, but the others used a variety of matrix techniques. Three of them were willing to restore ≥ 80% of cases, and 14 were willing to restore < 60% of cases. Nine respondents (17%) never used rubber dam. Eight of these were willing to restore ≥ 89% of cases and seven used all matrix techniques (individually and in combination).

### Regression Analyses

Details of the logistic regression analyses are shown in Table 3 and Appendix Tables A2 to A10 (with explanations).

**Table 3:** Cavity characteristics influencing the willingness to place a direct class II composite restoration

Independent cavity variable (predictor)	Odds ratio	P > z	95% confidence interval
Occlusal extension (reference none)	0.3	0.102	0.07–1.3
MOD or larger (reference not)	0.4	**0.004**	0.3–0.8
Broad (reference not)	0.4	**0.008**	0.2–0.8
Large bridging gap (reference not)	0.2	**0.000**	0.2–0.4
Root filled (reference not)	0.3	**0.000**	0.2–0.6
Deep sub-gingival (reference not)	0.5	**0.032**	0.3–0.9
Thin cusp wall(s) (reference not)	0.7	0.172	0.5–1.1
Constant	88.2	**0.000**	21.3–364.9
n = 855; Pseudo-R^2^ = 0.3; Bayesian Information Criterion = 772.3; MOD, mesio-occluso-distal.			

The inverse of the odds ratios presented in Table 3 represents that experts’ willingness to restore class II cavities with direct composite was statistically significantly:

4.2 times less likely when the interproximal “bridging” gap was large
3.1 times less likely when the tooth was root filled
2.4 times less likely when the box was broad
2.2 times less likely when the cavity extension was MOD or larger
1.8 times less likely when the margin(s) were deep sub-gingival
(thin cusp walls and occlusal extension were not statistically significant variables)

As examples, respondents willing to restore ≥ 80% presented cases with direct composite were much more likely to primarily restore previously restored teeth (12.0 times; *P* < 0.001), use increased curve circumferential matrices (7.6 times; *P* = 0.046), and much less likely to use rubber dam (4.9 times; *P* < 0.001). Providing cuspal coverage when restoring a cavity was much more likely when it had occlusal extension than when it did not (36.8 times; *P* < 0.001), MOD extension or more (4.0 times; *P* < 0.001), a root filling (3.2 times; *P* = 0.001), and thin cusp wall(s) (2.8 times; *P* < 0.001). When restoring single-surface “claustrophobic” class II restorations, respondents were much more likely to use a sectional matrix alone (6.6 times; *P* < 0.001) and fill the whole cavity in one increment (3.7 times; *P* = 0.001). When restoring teeth with deep sub-gingival margins, respondents were much more likely to perform a papillectomy (43.1 times; *P* < 0.001), use increased curve circumferential matrices (9.7 times; *P* = 0.005), perform delayed wedging (2.8 times; *P* = 0.016) and light cure the first flowable composite increment (2.5 times; *P* = 0.002), and were much less likely to use a sectional matrix alone (2.7 times; *P* = 0.001). Similarly, when restoring teeth with broad boxes and large bridging gaps, respondents were much more likely to use increased curve circumferential matrices (9.8 times; *P* = 0.003; 15.5 times; *P* < 0.001, respectively) and perform a papillectomy (4.3 times; *P* < 0.001; 2.7 times; *P* < 0.001, respectively), and less likely to use sectional matrices alone (4.9 times; *P* < 0.001; 3.7 times; *P* < 0.001). When restoring MOD or larger cavities, respondents were much more likely to use a transfer matrix (15.8 times; *P* = 0.005) and a snowplow (injection molding composite application technique) (3.3 times; *P* < 0.001) and less likely to use a sectional matrix alone (6.9 times; *P* < 0.001). When restoring teeth where an adjacent tooth was present, respondents were much more likely to use a sectional matrix alone (12.3 times; *P* < 0.001) and less likely to use saddle matrices (8.1 times; *P* = 0.001). Rubber dam use was much more likely when sectional matrices were used (12.5 times; *P* < 0.001) and was less likely in those willing to restore ≥ 80% of presented cavities (3.7 times; *P* < 0.001) or where there were adjacent cavities (3.4 times; *P* < 0.001)(Appendix Tables A2 to A10).

## DISCUSSION

There was a wide variation among experts in their willingness to restore varying presentations of class II cavities with direct composite. It is important to note that the question for each cavity did not ask if direct restoration was favored, just if it would be provided under any circumstances. The results obtained from the regression analyses are broadly as expected and therefore provide a level of face, external, and internal validity to the variable selection and models.

### Willingness to restore and material and technique use

This study provides evidence that varying presentations of class II cavities have differing complexities when restoring them with direct composite, and these affect many experts’ willingness to restore them. These complexifying factors, in order of importance, were: large bridging gaps (the distance between the periphery of the box and the maximum bulbosity of the adjacent tooth); presence of a root filling; broad cavities, where the cavity extent was MOD or greater; and if the cavity extended deep sub-gingivally. The thickness of the remaining cusps did not reach statistical significance. The likely rationale for the bridging gap being the most important factor is that achieving a good contact with the adjacent tooth using direct composite can be especially difficult. Getting a matrix to seal at the base while also having sufficiently aggressive emergence to cross the gap to the adjacent tooth can be challenging. Techniques using increased curve circumferential matrices can overcome this issue to a degree, especially when used alongside sectional matrices in two-stage techniques,^11,17^ but can be more time-consuming, technically complex, and require a larger inventory of equipment.

These data also show that when present, the complexifying variables affect materials and techniques used. For example, where cavities had deep sub-gingival margins, papillectomies were much more commonly used, which can allow effective isolation, matrixing, and wedging of deep cavities,^5^ as were increased curve circumferential matrices. These matrices were also much more frequently used in cavities with large bridging gaps and broad boxes. They were also much more commonly used by dentists who were willing to restore a large majority of the presented cases directly and were much less likely to use a rubber dam. Sectional matrix use alone and conventional rubber dam use were highly correlated, with 15% of respondents always using rubber dam and sectional matrices. The vast majority of these (but not all) were willing to restore less than 60% of presented cases directly. This suggests that for a significant minority, direct restorations were limited to simpler cavities, as commonly advocated in textbooks and expert guidance,^27,31,32^ when rubber dam and sectional matrices are easily used. Conversely, those willing to use alternatives to rubber dam and use multiple techniques (including curved circumferential matrices) were more likely to be willing to restore more complex cavities directly. There was, however, a large variation.

It cannot be said definitively that the respondents who would not restore the cavities directly would have restored them with indirect restorations, as this was not specifically asked, but this seems likely in a large majority of cases. There is a tension between the laboratory data, which often favor indirect restorations in terms of biomechanical behavior but have questionable clinical translation and/or significance, and the clinical survival data, which suggest an equivalence between large direct and indirect restorations.^7,8,15,24,27,30–33^ Textbooks and guidance also often advocate for indirect restorations in complex cavities,^7,27,30–33^ whereas a recent German S3 clinical practice guideline does not preclude direct composite.^34^ This conflicting data and guidance, alongside prescriptive technical guidance on placing direct restorations that recommend the use of sectional matrices and rubber dam isolation,^22^ the perceived technical difficulty and failure risk of restoring complex cavities directly, may influence expert attitudes and “willingness” to place complex direct restorations in practice.

Limited primary care National Health Service data shows that a majority of root-filled teeth treated in the UK were restored directly (with amalgam),^18,19^ possibly because of the much higher initial costs of indirect restorations. Robust health economic data are not available to assess comparative lifetime costs; however, this would likely vary based on patient characteristics, the operator, service provision, and remuneration in different health systems.

Previous related studies on willingness to restore with direct composite focused on missing cusps, with other potential complexifying factors not explored or varied. A broadly representative Norwegian study of general dentists found that 87% of respondents would restore a molar with one missing cusp with direct composite as their first choice, and this fell to 36% with the loss of two cusps and 7% with the loss of three.^16^ The reported proportion restoring with composite when two cusps were missing was much lower in a similar Kuwaiti study, and younger dentists and those working in the private sector preferred indirect restorations.^1^

### Cuspal Coverage

There was significant variation in respondents’ provision of cuspal coverage, though nearly all indicated that they would provide it in at least some cavities. The two respondents who never provided cuspal coverage were only willing to restore less than 60% of the presented cases, so they were not providing direct restorations for more compromised teeth. Cuspal coverage was associated with cavities having occlusal extension (compared with not), MOD extension or more, a root filling, and thin cusp walls. Recent guidance generally recommends cuspal coverage restorations for root-filled teeth, with one document not specifying the use of direct or indirect materials,^23^ and another focusing more on indirect restorations 7.

There is no obvious consensus on the provision of cuspal coverage in vital teeth, as there is a general absence of good-quality clinical data, and *in vitro* studies offer conflicting results in terms of survival and fracture strength when covering a cusp or not under differing loading conditions.^7,12,13,30^ It does seem that covering a cusp may result in improved longevity of the restored tooth unit with cyclical loading, but perhaps deeper, more catastrophic fractures occur at failure (often of an already compromised tooth, however).^10,12^ Expert guidance is therefore often largely based on *in vitro* data,^2,7,27,30^ which is not always in agreement and has uncertain clinical relevance.

### Implications

The findings of this study present significant implications for clinical trials, as many complexifying cavity characteristics have not previously been recorded or controlled for. This can have implications on the technical difficulty of performing a direct class II composite restoration and, therefore, potentially restorative outcomes. These characteristics should therefore be recorded (ideally using intraoral scanning) and controlled for, or considered in the trial design and randomization into treatment groups. It also has significant implications when analyzing and interpreting retrospective or non-randomized restoration survival data, as indication bias is unavoidable in these studies. This relates to how the differing choice of direct or indirect restoration (or perhaps extraction) for a given cavity presentation will hugely influence survival data for the various restorations provided. Controlling for complexifying factors, alongside operator and patient factors, would help to mitigate this.

The study has implications for how clinicians are paid for restoring different presentations of cavities, as fees are commonly set by the surfaces involved, especially in national health systems where fees cannot be changed.^4^ This study has shown that many factors other than the surfaces involved are more important in terms of complexity, which may then affect the materials and techniques used. Complex cavities potentially require less commonly available equipment (eg, curved circumferential matrices) and/or more complex processes, eg, multiple matrix bands, which may be more time-consuming and expensive.^4^ This could influence practitioners’ willingness to restore more complex presentations with direct restorations (alongside their sentiments on the appropriateness of using composite in these situations, among others, for example). Private practitioners may charge by time, which could overcome this concern to a degree; however, it may favor the prescription of more expensive indirect restorations. In most health systems or services, indirect restorations are much more expensive than direct restorations, with a proportion of patients, especially those of low socioeconomic status, likely unable or unwilling to afford them. The differing thresholds for willingness to place large direct restorations demonstrated in this study could therefore disproportionately affect less affluent patients. This might influence their choice to extract teeth and could potentially exacerbate existing health inequalities.^4^ Incentivizing the provision of complex direct restorations through additional remuneration may help with this.

The current work has significant implications for clinician education and how some of the lesser-known or more complex techniques used by several practitioners are integrated into undergraduate curricula and postgraduate training. If dentists have been trained in these techniques to pragmatically and predictably place adequate direct restorations, this may result in more general dentists being comfortable in selecting or recommending a direct instead of an indirect technique, which might be beneficial in terms of being more minimally invasive as well as limiting the costs and time burden of dental care. This could potentially make dentistry more accessible for those of lower socioeconomic status, allowing them to maintain teeth for longer, though no good-quality health economic data on this exist.

Beneficial future research could include the collection of clinical observational data correlating stated willingness to restore with actual treatment choices, broader international samples including non-experts, and further work on patient-centered outcomes and values alongside health economic evaluations.

A follow-up, related paper will examine the general use of materials and techniques for class II restorations by clinicians with expertise and synthesize the whole body of work to provide guidance on the pragmatic restoration of complex class II cavities with direct composite. This aims to provide general dentists with easily applicable techniques to conserve teeth in a cost-effective way that is accessible to all.

### Limitations

There are various sources of errors and biases that could have affected the results. The study is cross-sectional and hypothetical. The sample is based on a convenience sample of clinicians deemed to have expertise (ascribed by the core group) in direct restorations, which is therefore at risk of selection bias. It is therefore not representative of the broader dental community, with questionable generalisability. Respondents were working in different healthcare systems, often with different pressures, demands, remuneration, and patient characteristics and values, all of which likely affect treatment planning and provision. There are also concerns over self-reporting, small sub-group sizes, and potentially unbalanced presentation of variables in the vignettes. Adding more vignettes could potentially have overcome this, but the survey was long, commonly taking over an hour to complete. This likely contributed to a small proportion of respondents failing to complete the survey, and there is a risk of fatigue bias. However, the response and completion rates were high, and the results all have face validity, providing confidence in the methods and analyses used.

## CONCLUSIONS

Cavity complexity strongly influences experts’ willingness to place direct composite restorations. Complexifying factors were: large bridging gap, presence of a root filling, broad cavities, where the cavity extent was MOD or greater, and if the cavity extended deep sub-gingivally. Some experts always used rubber dam isolation and sectional matrices and never covered cusps, but were only willing to restore lower complexity cavities with direct composite. Experts with a higher willingness to treat complex cases tend to adopt multiple techniques, including the use of curved circumferential matrices and alternatives to rubber dam isolation. Understanding these patterns can inform guideline development, training, and shared decision-making, potentially helping to maintain tooth structure and reduce inequalities where indirect care is less accessible.

### Acknowledgments

Thanks to Dentsply Sirona (DS) (Dentsply DeTrey, Konstanz, Germany) for supporting the attendance of OB, TA, and BL at the meeting of EFCD and ORCA, July 2023, in Egmond aan Zee (NL), and providing the contracted version of SurveyMonkey, and to Chris O’Connor, Alan Burgin, and Neeraj Diddee for providing vignette case photographs. RF, OB, DW, and TA have received lecture honoraria from DS (and other companies in the dental industry), RF has received grants from DS and AKL, DW, TA, and RF are members of the DS restorative advisory board.
